# Conformational control of two-dimensional gold nanoparticle arrays in a confined geometry within a vesicular wall

**DOI:** 10.1038/s41598-022-08607-0

**Published:** 2022-03-16

**Authors:** Jong Dae Jang, Hyuk-Jin Seo, Young-Jin Yoon, Soo-Hyung Choi, Young Soo Han, Tae-Hwan Kim

**Affiliations:** 1 Neutron Science Division, Korea Atomic Energy Research Institute, 1045 Daedeok-daero, Yuseong-gu, Daejeon, 34057 Republic of Korea; 2 Research Center for Advanced Nuclear Interdisciplinary Technology, Jeonbuk National University, 567 Baekje-daero, Deokjin-gu, Jeonju, Jeollabuk-do 54896 Republic of Korea; 3 Department of Applied Plasma and Quantum Beam Engineering, Jeonbuk National University, 567 Baekje-daero, Deokjin-gu, Jeonju, Jeollabuk-do 54896 Republic of Korea; 4 Department of Chemical Engineering, Hongik University, 94 Wausan-ro, Mapo-gu, Seoul, 04066 Republic of Korea; 5 Department of Quantum System Engineering, Jeonbuk National University, 567 Baekje-daero, Deokjin-gu, Jeonju, Jeollabuk-do 54896 Republic of Korea; 6 High-Enthalphy Plasma Research Center, Jeonbuk National University, 546 Bongdong-ro, Bongdong-eup, Wanju-gun, Jeollabuk-do 55317 Republic of Korea

**Keywords:** Materials science, Nanoscience and technology

## Abstract

The two-dimensional (2D) assembly of gold nanoparticles (AuNPs) in a confined geometry is a rare phenomenon that has not been experimentally verified for complex systems. In this study, this process was investigated in detail using two types of block copolymers with hydrophobic and hydrophilic blocks and a series of AuNPs of three different sizes protected by hydrophobic ligands. In aqueous solutions, the selected block copolymers self-assembled into vesicular nanostructures with a hydrophobic domain in the wall, which functions as a confined geometrical space for hydrophobic AuNPs (i.e., it exerts a confinement effect and restricts the movement of AuNPs). Small-angle X-ray scattering studies revealed that AuNPs of different sizes assembled differently in the same confined geometry of the vesicular wall. In addition, optimal conditions for the formation of a regular NP array in the hydrophobic domain were determined. The AuNPs successfully self-assembled into a regular 2D lattice structure, forming a shell around the vesicle, when their size matched the thickness of the hydrophobic domain of the vesicular nanostructure. This study provides guidelines for the fabrication of nanoparticle arrays with controlled structures, which could enhance the functionality of materials and their physical properties.

## Introduction

In nature, the distribution of atoms and molecules are strongly affected by ambient conditions, such as pressure and temperature. However, most materials without any perturbation are characterized by their volumes, according to the ideal gas law^1–3^. Hence, a dilute nanoparticle suspension, which does not have a potential contributing to the interaction, can be regarded as an ideal gas on the nanoscale. In such a suspension, since the nanoparticles do not interact with each other, they are homogeneously distributed without making an aggregation or orientation^4–7^. Except their uniform dispersion (similar to that of the ideal gas molecules) and the absence of a volumetric effect, however, it is still unclear how the phase behavior and location of nanoparticles in a specific (small or confined) volume transforms. Therefore, to make sure the specific volumetric effect in the formation of nanoparticle lattice structure, it needs the study on the self-assembly of the dilute nanoparticles in the confined geometry system. For this purpose, gold nanoparticles (AuNPs) with a hydrophobic surface (which can be easily controlled by the capping agent^7–10^) and polymer vesicles were utilized in this work. Typical amphiphilic polymers form spherical micelles in water; however, polymers with long hydrophobic chains can easily form vesicular structures^11–13^. In general, amphiphilic block copolymers containing long hydrophobic chains form vesicles^13,14^ bound by a membrane composed of three domains (i.e., distinct hydrophilic–hydrophobic–hydrophilic regions). In the vesicular membrane, the hydrophobic domain can serve as a confined geometry that can be easily controlled by varying external conditions. The moisture content of vesicular structures is strongly affected by various parameters such as the presence of additives, temperature, solution pH, and concentration, and an increase in the degree of hydrophobicity of the vesicles leads to their dehydration^13^. Dehydrated vesicles have a denser membrane structure, and the hydrophobic region of the membrane represents a confined geometry that can entrap various materials (such as AuNPs) through hydrophobic interactions^15,16^. Thus, the present study was conducted to investigate the behavior of a nanomaterial in the narrow or confined nanospace of a vesicular membrane.

To expand the bio-applications of vesicular nanostructures, polyethylene glycol (PEG) polymer is generally selected as the hydrophilic block of block copolymers to form an outer bio-compatible vesicle layer. This is because PEG has been approved as a bio-compatible material by the Food and Drug Administration (FDA) of the United States. Various research groups have investigated possible applications of PEG and PEG-based block copolymers as biomaterials^17–19^. Previously, diblock copolymers consisting of poly(ethylene glycol) methyl ether (mPEG) and poly(isopropyl glycidyl ether) (PiPGE) blocks (denoted as P(EG-*b*-iPGE)), which form vesicles, have been synthesized through anionic ring-opening polymerization (AROP)^16^.

When a particle is entrapped in a two-dimensional (2D) hydrophobic layer of a block copolymer (i.e., 2D confined geometry), its degree of freedom is restricted to two (up–down) directions by the volume of the confining region. Controlling the hydrophilic mass fraction (*f*) of the block copolymer can effectively cause a change in the phase and vesicular wall layer thickness of the P(EG-*b*-iPGE) complex (note that the phase of the complex changes in the lower range of temperature compared to that in ambient conditions)^16^. Thus, the *f*-control method may be used to adjust the geometry and hydration degree of the resultant phase^13^. To vary the membrane layer thickness of the vesicle, we used two types of P(EG-*b*-iPGE) diblock copolymers with molecular weights of 5 k–5 k and 0.75 k–2.35 k^15^. The larger diblock polymer (molecular weight: 5 k–5 k) formed vesicles at 0.25 < *f* < 0.45, but produced spherical nanostructures at *f* = 0.5. However, when the smaller P(EG-*b*-iPGE) polymer (molecular weight: 0.75 k–2.35 k) was injected into a solution containing the former P(EG-*b*-iPGE) polymer, the formation of vesicles at 0.25 < *f* < 0.45 was promoted.

In our previous study, we synthesized an AuNP–polymer vesicle hybrid and confirmed the formation of an AuNP single-layered structure in the hydrophobic layer of the vesicle^15,16^. However, we did not elucidate the formation mechanism of an array of AuNPs around the vesicle, which was strongly affected by the thickness of the lamellar wall of the vesicle (the squeezing and thinning of the membrane walls determined the size of the AuNPs that were entrapped). Thus, the flexibility of the lamellar wall facilitated the entrapment of hydrophobic AuNPs in the hydrophobic region. The controlled hydrophobic surface of AuNPs also made a good stability to aggregate themselves in the hydrophobic region.

To determine the degree of order in the AuNP array formed in the vesicle layer, small-angle X-ray scattering (SAXS) measurements were performed in this work. In addition, to study the effect of the AuNP size on its fate in the confined volume of the vesicle layer, AuNPs with different diameters of 2, 4, and 6 nm were used. As the AuNP size changed, the resulting SAXS intensities revealed different degrees of interparticle interaction in the self-assembled AuNP array in the membrane of the vesicle. The Bragg reflections of the produced AuNP arrays were indexed with the peak ratios of a centered rectangular structure. Furthermore, the interparticle interactions in the AuNP arrays with different particle sizes were compared by analyzing the intensities of the corresponding SAXS peaks. The self-assembly of the AuNPs depended on the volume of the hydrophobic region formed by the amphiphilic polymer aggregates. In addition, as the AuNPs had hydrophobic surfaces and they exhibited strong interparticle interactions, the overall shape of the polymer vesicle was affected by the hydrophobic volume of the AuNP-polymer hybrid. This result may be an excellent example of the formation of an array of AuNPs in a confined geometry using particles of different diameters. Furthermore, this study provides insights on nanomaterial arrangements under the effect of a confined geometry. The synthesized AuNP–polymer vesicles can potentially be utilized as nanocarriers for drug delivery or nanodevices for electron exchange.

## Results and discussion

The effect of confined geometry on AuNPs leads to various outcomes, including the formation of AuNP arrays. To control the volume of the confined nanospace, that is, the hydrophobic domain in a vesicle, we varied the hydrophobic chain volume of the diblock copolymer. On the one hand, increasing this volume increases the thickness of the hydrophobic layer in the vesicle. On the other hand, increasing the number of hydrophobic chains is equivalent to decreasing the hydrophilic mass fraction (*f*). The hydrophobicity of the vesicle promotes the dehydration of the vesicle membrane, thereby reducing its thickness. Note that the thickness of the vesicle layer is highly sensitive to even small changes in the experimental conditions. In this study, the vesicle was formed by maintaining *f* in the range of 0.25 < *f* < 0.45. In this study, two types of P(EG_x_-*b*-iPGE_y_) diblock copolymers with *f* = 0.50 (P(EG_113_-*b*-iPGE_45_) (denoted as P(113-45)) and *f* = 0.25 (P(EG_16_-*b*-iPGE_21_) (denoted as P(16-21)) were mixed to adjust the *f* value of the resultant mixture to form vesicles in an aqueous solution. The two copolymers were synthesized via AROP (Supplementary Fig. S1) and characterized by nuclear magnetic resonance (NMR) and gel permeation chromatography (GPC). The block copolymers, P(113-45) and P(16-21) had molecular weights of 5 k–5 k and 0.75 k–2.35 k, respectively, and their polydispersity indexes $(\overline{{M}_{w}}/\overline{{M}_{n})}$ were 1.04 and 1.05, respectively. Considering our previous study^16^, we expected that the vesicles formed using a mixture of these two diblock copolymers contained a hydrophobic layer with a thickness of 4–5 nm sandwiched between hydrophilic layers; the calculated stretched lengths of the iPGE_y_ block at y = 45 and y = 21 were 27.7 and 13.0 nm, respectively. Thus, the thickness of the hydrophobic layer of the vesicle was optimized to be comparable to the AuNP diameters (2, 4, and 6 nm) used for entrapment, and thus have a sufficiently confined volume that effectively inhibits their movement. The AuNPs used in this study had average diameters of 2.15 nm (± 0.42 nm, polydispersity index: 0.11), 3.85 nm (± 0.32 nm, polydispersity index: 0.08), and 6.27 nm (± 0.34 nm, polydispersity index: 0.09), as confirmed by transmission electron microscopy (TEM) of the particles (Fig. S2).

**Figure 1:**
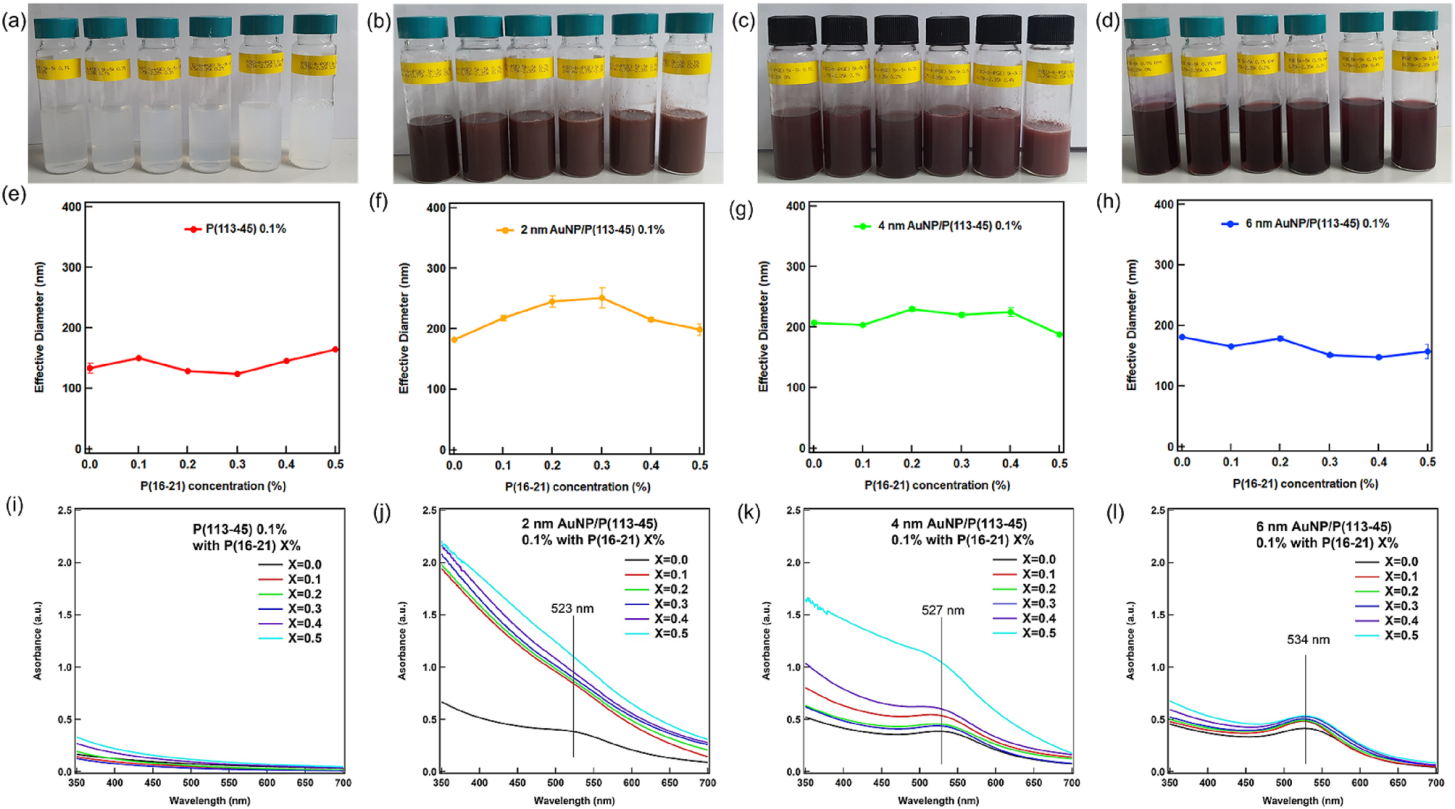
Basic characteristics of the diblock polymer mixtures. Visual inspection of the P(113-45)/P(16-21) mixtures with (**a**) no AuNPs, (**b**) 2 nm AuNPs, (**c**) 4 nm AuNPs, and (**d**) 6 nm AuNPs. DLS results of the P(113-45)/P(16-21) mixtures with (**e**) no AuNPs, (**f**) 2 nm AuNPs, (**g**) 4 nm AuNPs, and (**h**) 6 nm AuNPs. UV–Vis spectra of the P(113-45)/P(16-21) mixtures with (**i**) no AuNPs, (**j)** 2 nm AuNPs, (**k**) 4 nm AuNPs, and (**l**) 6 nm AuNPs.

To form various vesicular nanostructures, the P(113-45)/P(16-21) and AuNP–P(113-45)/P(16-21) mixtures were diluted with water and visually inspected to evaluate their ability to form a vesicle (Fig. 1a–d). As the P(16-21) concentration was increased with respect to that of P(113-45), the turbidities of the mixtures increased owing to strong reflections caused by the Tyndall effect, indicating that large self-assembled colloidal structures were formed in the mixture^20^. According to previous studies, the morphology of the vesicular nanostructure strongly influences the Tyndall effect^13^. The particle sizes in the obtained mixtures determined by dynamic light scattering (DLS) were 120–250 nm (Fig. 1e–h), which represent standard micelle sizes and also indicate the presence of much larger structures. The larger structures could be considered to be vesicular structures because their geometry was predicted by analyzing the hydrophilic moieties of the polymer. UV–visible (vis) spectra were analyzed to study the changes in the intrinsic properties of the AuNPs in the polymer mixture by investigating their surface plasmon resonance (SPR) characteristics (Fig. 1i–l)^21^. If the UV–vis peak position does not change with increasing P(16-21) concentration, it can be concluded that the AuNPs remained separate without forming aggregates/assemblies^22,23^. Therefore, the morphology of the AuNP–polymer mixture was investigated by conducting DLS and UV–vis measurements. When the P(113-45) diblock copolymer (*f* = 0.50) was blended with the P(16-21) diblock copolymer (*f* = 0.25), as shown in Fig. 1a–d, the degree of turbidity of the aqueous mixture increased with increasing concentration of the P(16-21) block copolymer because the Tyndall effect is sensitive to the size of the nanostructures formed by their assembly. The results of DLS measurements confirmed that the sizes of the produced aggregates were in the range of 123.9–250.8 nm. The sizes changed as the polymer aggregate geometry changed with the increase in the P(16-21) concentration (Fig. 1e–h). Meanwhile, the particle size of the P(113-45)/P(16-21) mixture was variable and could not be determined by DLS.

The SPR peak positions of the AuNPs with sizes 2, 4, and 6 nm were 523, 527, and 534 nm, respectively. The obtained UV–Vis spectra of the AuNP–polymer mixtures, which showed an increase in the corresponding SPR peak intensities, confirmed the presence of AuNP aggregates in the studied mixtures (Fig. 1i–l). In general, the UV–Vis absorbance increased with an increase in the P(16-21) concentration. However, the SPR peak positions of the AuNPs did not depend on the P(16-21) concentration, suggesting that the AuNPs did not coalesce. Meanwhile, the observed change in absorbance with the change in the size distribution of AuNPs suggests that their intrinsic properties can be adjusted by varying the particle size^22^.

**Figure 2:**
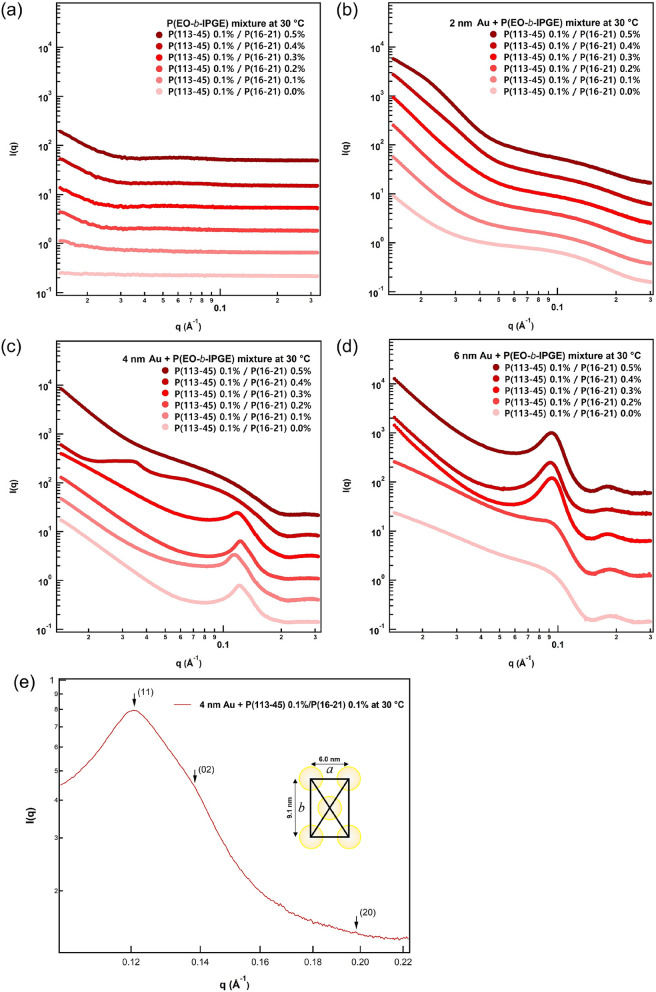
SAXS intensities of P(113-45)/P(16-21) mixtures with (**a**) no AuNPs, (**b**) 2 nm AuNPs, (**c**) 4 nm AuNPs, and (**d**) 6 nm AuNPs. (**e**) Bragg reflection of a P(113-45)/P(16-21) mixture with 4 nm AuNPs recorded at 30 °C.

Based on the results of visual inspection and DLS measurements, we anticipated the formation of vesicular nanostructures in the AuNP–P(113-45)/P(16-21) mixtures. However, the nature of AuNP organization could not be determined from UV–vis and DLS data. Therefore, SAXS measurements were performed both at ambient temperature (30 °C; Fig. 2) and in the range of 30–60 °C with 5 °C increments (Figs. S3–S6). Dilute P(113-45)/P(16-21) mixtures provided a very low X-ray contrast in the investigated temperature range (Fig. 2a). However, most of the P(113-45)/P(16-21) mixtures containing AuNPs yielded strong SAXS signals with Q^−2^ behaviors in the low Q region (< 0.03 Å^−1^), which confirmed the formation of vesicular nanostructures^13–16^.

The SAXS intensities varied considerably with the P(16-21) concentration, which could be attributed to the formation of different AuNP array structures depending on the AuNP size under the effect of the confined geometry of the vesicle wall. The observed *q*^−2^ behavior of the SAXS intensity likely resulted from intraparticle interference in the layered structure of the self-assembled AuNPs, reflecting the high scattering contrast of the AuNPs. In Fig. 2b, all SAXS profiles of the 2 nm AuNP–P(113-45)/P(16-21) exhibited *q*^−2^ behavior in the low *q* region, but exhibited no interaction peak. Therefore, considering the thickness of the hydrophobic region of the assembled block copolymer and AuNP size, it can be inferred that AuNPs formed a single layer with an irregular structure in the confined geometry. As shown in Fig. 2c, the SAXS profiles of the 4 nm AuNP–P(113-45) 0.1%/P(16-21) 0.0–0.3% samples exhibited strong interaction peaks and *q* ^−2^ behavior in the low *q* region. Hence, it can be concluded that 4 nm AuNPs formed a single layer with a specific arrangement of AuNPs (i.e., a close-packed AuNP array) in the confined geometry. However, in the case of the 4 nm AuNP–P(113-45) 0.1%/P(16-21) 0.4–0.5% samples, the P(113-45)/P(16-21) vesicular structure presumably ruptured, and the P(113-45)/P(16-21) polymer aggregate was likely transformed to another structure. The corresponding SAXS profiles in Fig. 2d show strong signal intensities without sharp peaks. For the 6 nm AuNP–P(113-45) 0.1%/P(16-21) 0.0–0.2% samples, the slopes of the SAXS profiles exhibited a trend that lies between *q*^−2^ and *q* ^−1^ behaviors, indicating a mixed phase with cylindrical and vesicular structures. Meanwhile, for the 6 nm AuNP–P(113-45) 0.1%/P(16-21) 0.3–0.5% samples, the slope of the SAXS profiles demonstrated a nearly ideal *q* ^−2^ behavior, suggesting that AuNPs formed a single layer with an unspecified arrangement in the confined geometry. Note that because the thickness of the hydrophobic layer of the vesicle was smaller than the diameter of the AuNPs, it was difficult to fit the 6 nm AuNPs into the confined geometry (hydrophobic region), unlike the 2 and 4 nm AuNPs. Therefore, some 6 nm AuNPs invaded the hydrophilic region of the vesicle to avoid direct contact with water molecules, thus occupying additional space in the vesicular structure.

In this study, the 4 nm AuNP–P(113-45) 0.1%/P(16-21) 0.1% sample had the optimal configuration of assembled AuNPs. The corresponding SAXS profile contained three peaks at *q* = 0.121, 0.138, and 0.199 Å^−1^ that could be indexed to the (11), (02), and (20) Bragg reflections of a centered rectangular structure, respectively (Fig. 2e). For a centered rectangular structure with 2D lattice constants of *a* = 6.0 nm and *b* = 9.1 nm, the scattering peaks would be located at ${q}_{\mathrm{hk}}=2\pi \sqrt{{\left(h/a\right)}^{2}+{\left(k/b\right)}^{2}}$ (*a* and *b* are the 2D lattice parameters)^16^. The results indicate that a regular 2D array of individual AuNPs with a diameter of 4 nm was formed in the 4 nm AuNP–P(113-45) 0.1%/P(16-21) 0.1% sample.

**Figure 3:**
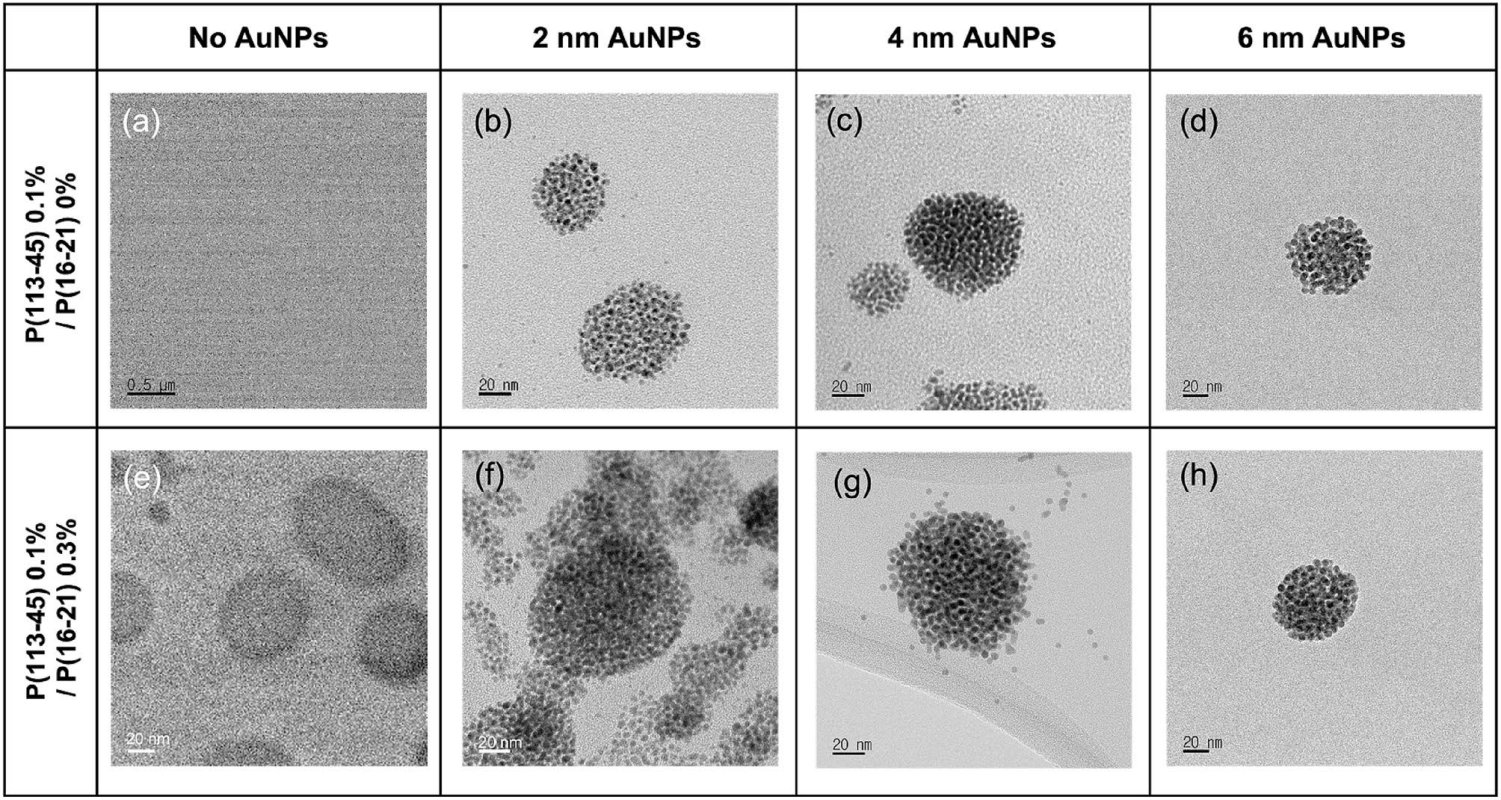
TEM images of the P(113-45) 0.1%/P(16-21) 0% mixture with (**a**) no AuNPs, (**b**) 2 nm AuNPs, (**c**) 4 nm AuNPs, and (**d**) 6 nm AuNPs, and P(113-45) 0.1%/P(16-21) 0.3% mixture with (**e**) no AuNPs, (**f**) 2 nm AuNPs, (**g**) 4 nm AuNPs, and (**h**) 6 nm AuNPs.

To study the formation of the AuNP–P(113-45)/P(16-21) self-assembled structure, TEM observations were performed (Fig. 3). In the obtained TEM images, the contrast of AuNPs is dominant, whereas the contrast of the polymer vesicle in the mixture is very low (Fig. 3a,e). The TEM images of the AuNP–P(113-45)/P(16-21) samples show spherical structures composed of small particles (AuNPs) (Fig. 3b–d,f–h). The results indicate that the hydrophobic AuNPs were located in the hydrophobic region of the P(113-45)/P(16-21) complex to avoid water molecules. Considering the polymer forms a vesicular shape (Fig. 3e, therefore, AuNPs formed a layer with a single NP thickness in the membrane of the vesicular nanostructure. This interpretation is consistent with the results of small angle neutron scattering (SANS) analysis (Fig. S7).

**Figure 4:**
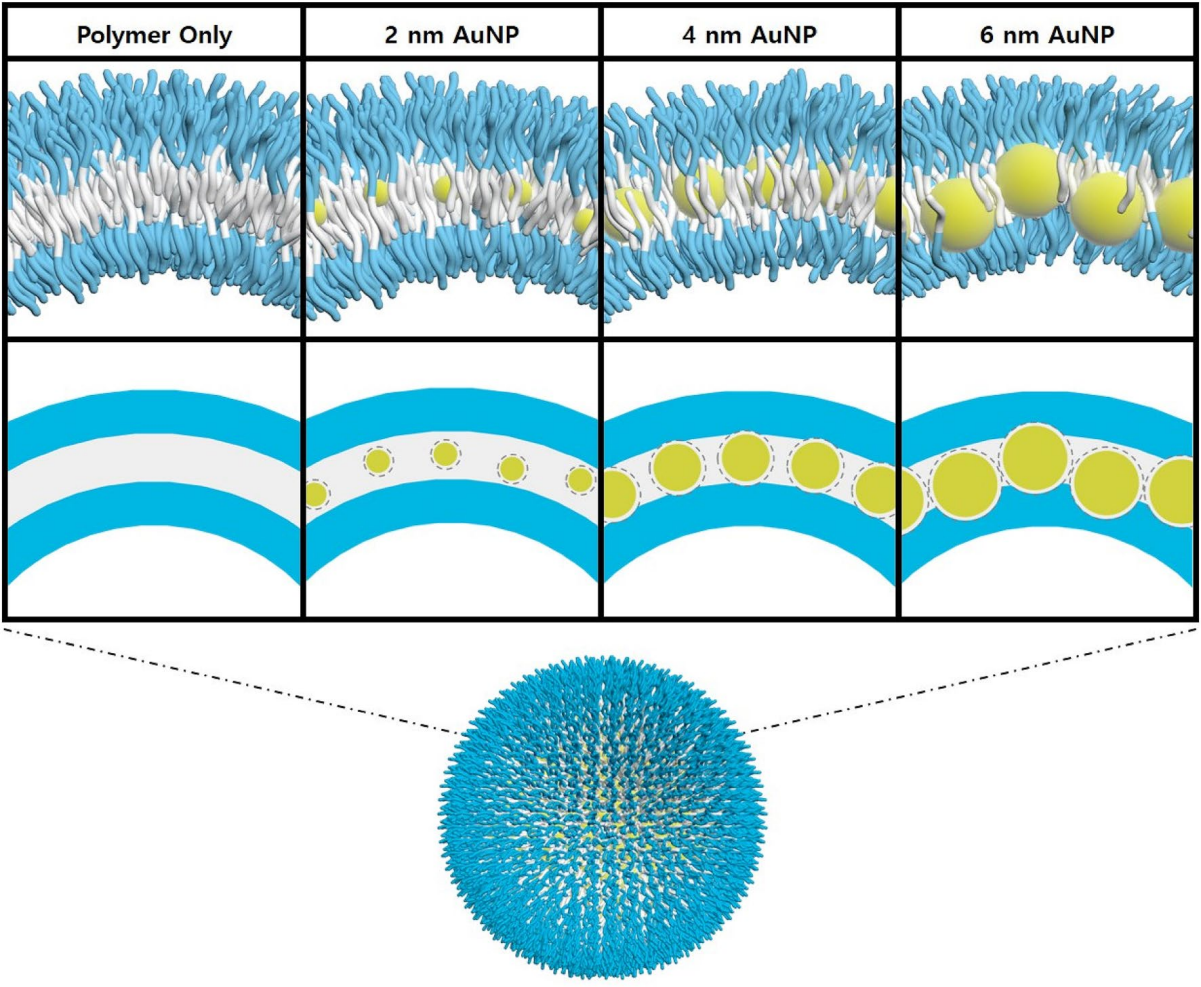
Schematic of the vesicular nanostructures of the AuNP–P(113-45)/P(16-21) mixtures.

In fact, the TEM images of the mixture without AuNPs exhibited no distinct features because of the low contrast of the polymer mixture with a very small polymer concentration of 0.1% (Fig. 3a). However, when the polymer concentration was increased to 0.4%, a vesicular shape was observed (Fig. 3e). On the other hands, as the polymer vesicle entrapped AuNPs, the self-assembled nanostructure of the AuNP–P(113-45)/P(16-21) mixture with a spherical shape could be readily observed in TEM. AuNPs were expected to form a single layer in the vesicular nanostructure based on the *q*^−2^ dependence of the SANS and SAXS intensity. In addition, to determine the degree of interparticle interaction in the AuNP arrays produced under different conditions, the temperature was varied; however, the resulting SAXS intensities changed only slightly and remained close to the value obtained at 30 °C. Therefore, the degree of interparticle interaction in the AuNP array was not significantly affected by temperature, but the morphology of the self-assembled structure could be controlled (Fig. S4–S6). A schematic of the self-assembled AuNP–P(113-45)/P(16-21) nanostructure is presented in Fig. 4 based on the SAXS and TEM data. The AuNPs are incorporated in the thin hydrophobic layer of the vesicle of the P(113-45)/P(16-21) polymer mixture. The formation of the AuNP array is affected by the hydrophobic interaction of the AuNP surface with the hydrophobic layer and the confined geometry effect of the vesicular structure of the P(113-45)/P(16-21) mixture. In the self-assembled structures of the amphiphilic P(EG_x_-*b*-iPGE_y_) block copolymers, the PiPGE blocks generate hydrophobic regions to avoid direct contact with water, thus increasing the entropy of the system, and AuNPs were incorporated in this region. According to the DLS and TEM data, the confined geometry effect of the vesicle and the interaction between the hydrophobic layer and hydrophobic AuNP act simultaneously, resulting in a regular array of AuNPs. However, because the number of degrees of freedom (up–down) of the AuNPs is restricted to two by the confined geometry, the AuNP array becomes irregular.

## Conclusion

In this study, we investigated the effect of the confined geometry of the hydrophobic domain in the vesicular wall composed of a P(113-45)/P(16-21) mixture on the formation of an AuNP array. The AuNPs self-assembled into a regular 2D array in the confined nanospace of the vesicular wall, representing a rare phenomenon that occurs when the size of the confined geometry matches with that of the entrapped material. AuNPs spontaneously self-assembled in the P(113-45)/P(16-21) nanostructure; however, the confined geometry effect of the P(113-45)/P(16-21) vesicle controlled the arrangement of the AuNPs. When the degrees of freedom (up–down) of AuNPs are governed optimally by the confined geometry, AuNPs are arranged regularly. However, when they are weakly or strongly governed by the confined geometry, the AuNP array becomes irregular. In the 4 nm AuNP–P(113-45)/P(16-21) mixture (representing the optimal AuNP array formation condition), the thickness of the hydrophobic layer of the vesicle and the average AuNP diameter were similar. Hence, the confined geometry effect produced a regular array with single NP thickness. In the 2 nm AuNP–P(113-45)/P(16-21) case, the confined geometry effect was relatively weak, and the AuNPs formed a single layer without regular arrangement in the hydrophobic region. In the 6 nm AuNP–P(113-45)/P(16-21) mixture, the average diameter of the AuNPs was larger than the thickness of the hydrophobic layer of the vesicle, and the effect of the confined geometry was extremely strong. As a result, some AuNPs invaded the hydrophilic region of the vesicle to remain in the confined geometry. Therefore, the particles occupying a confined area can spontaneously form a regular array under certain conditions (this phenomenon was observed for the 4 nm AuNP–P(113-45)/P(16-21) mixture). The AuNP-polymer mixtures may provide an advantage of Fe^3+^ detection due to their unique structure with a large active area. This study demonstrates a promising route for the fabrication of materials with new functionalities and enhanced physical properties.

## Materials and methods

### Materials

The block copolymers were synthesized using the following compounds: poly(ethylene glycol)methyl ether (mPEG; molecular weights: Mn = 5000 and 750 g mol^−1^), isopropyl glycidyl ether monomer (iPGE; molecular weight: Mw = 116.16 g mol^−1^, 98%), potassium cube (in mineral oil, 99.5%), naphthalene (99%), butyl magnesium chloride solution in tetrahydrofuran (THF; 2 m), sec-butyl lithium solution in cyclohexane (1.4 m), and anhydrous hexane (99%) were purchased from Sigma–Aldrich (USA). THF (anhydrous, 99.9%) was obtained from Junsei (Japan). Anhydrous methanol (99.9%) was acquired from Alfa Aesar (USA). THF and iPGE were purified before use. AuNPs were synthesized using chloroauric acid (HAuCl_4_, 99.9985%) purchased from Strem Chemical, Inc. (USA), borane tert-butylamine complex (TBAB, 97%) purchased from Sigma–Aldrich, and oleylamine (70%) and 1,2,3,4-tetrahydronaphthalene (tetralin, 99%) purchased from Acros Organics (USA).

### Purification of iPGE and THF

The iPGE monomer was purified by stirring it with butyl magnesium chloride solution (2.0 m) for 30 min and then degassing by three freeze–pump–thaw cycles in a vacuum system. To remove impurities from THF, it was reacted with a sec-butyllithium solution (1.4 m) for 30 min under vigorous stirring followed by three freeze–pump–thaw cycles.

### Synthesis of block copolymers

P(EG_x_-*b*-iPGE_y_) diblock copolymers were synthesized via AROP. As this reaction is extremely sensitive to O_2_ and H_2_O molecules, all synthesis steps were conducted in vacuum. The mPEG polymer blocks used for preparing P(EG_113_-*b*-iPGE_45_) (5 g) and P(EG_16_-*b*-iPGE_21_) (1 g) were purified in a vacuum reactor at 45 °C and continuously stirred. Next, potassium naphthalenide initiator solution (5 mL, 0.4 m) was injected into the reactor followed by the direct injection of purified THF (10 mL). After the initiator injection, the mPEG solution turned dark-green, and the initiation reaction was performed for 30 min. Subsequently, the iPGE monomers (20 and 16 g) were added to the mPEG polymer solutions used for preparing P(EG_113_-*b*-iPGE_45_) and P(EG_16_-*b*-iPGE_21_), respectively. The propagation process was performed for 20 h until the solution turned light-brown followed by the termination of the reaction with anhydrous methyl alcohol. The final products were precipitated using hexane, and residual solvents were subsequently evaporated in vacuum for a day. The maximum conversion ratio of mPEG to the P(EG_113_-*b*-iPGE_45_) and P(EG_16_-*b*-iPGE_21_) block copolymers was 70%.

### Synthesis of AuNPs

AuNPs were synthesized using the Brust method^24,25^. The Au precursor solution consisting of HAuCl_4_ (0.4 g), tetralin (40 mL), and oleylamine (40 mL) was first stirred in a flask (500 rpm) for 10 min. In parallel, a reducing solution consisting of TBAB (0.32 g), tetralin (4 mL), and oleylamine (4 mL) was ultrasonicated in a vial for 10 min at 25 °C, and then injected into the Au precursor solution. The reaction mixture turned red immediately. The mixture was further stirred for 1 h at 45, 25, or 5 °C to synthesize AuNPs with sizes of 2, 4, or 6 nm, respectively. The final suspension was mixed with ethanol for AuNP precipitation, and the obtained AuNPs were collected by centrifugation for 8 min at 3000 rpm and then redispersed in fresh hexane (5 g/L). The size of prepared AuNPs was confirmed by TEM and SAXS measurements.

### Preparation of AuNP/polymer mixtures

The P(EG_x_-*b*-iPGE_y_) diblock copolymers with molecular weights of 5 k–5 k (P(EG_113_-*b*-iPGE_45_)) and 0.75 k–2.35 k (P(EG_16_-*b*-iPGE_21_)) were dispersed in water and the resulting mixtures were diluted to facilitate the observation of the micelle formation process by eliminating interparticle interferences. The concentration of the P(EG_113_-*b*-iPGE_45_) block copolymer was fixed at 0.1 wt.%, and that of the P(EG_16_-*b*-iPGE_21_) block copolymer was gradually increased at increments of 0.1 wt.% (0, 0.1, 0.2, 0.3, 0.4, and 0.5 wt.%) to control the geometry of the prepared samples in H_2_O (4 mL). The obtained polymer mixtures in water were divided into two parts, and were subsequently injected with AuNPs dispersed in 0.4 mL of hexane (AuNP concentration: 5 g/L) prepared in 2 mL H_2_O. Subsequently, the AuNPs were desolvated and dispersed in H_2_O by sonication for 8 h.

### UV–Vis measurements

UV–Vis absorbance studies were performed using a UV–Vis spectrophotometer (LAMBDA 950, Perkin Elmer Instruments Corporation). Absorbance spectra of AuNP/polymer mixtures in quartz cuvettes (path length: 10 mm) were recorded in the wavelength range of 190–3000 nm at 25 °C. The absorbance in the critical wavelength range of 350–750 nm was analyzed.

### DLS measurements

DLS measurements were conducted using a particle size analyzer (*λ* = 659 nm, ZetaPLUS, Brookhaven Instruments Corporation, USA) to determine the dependence of the micellar hydrodynamic radius (R_h_) at 25 °C. The DLS intensity autocorrelation function was obtained by a cumulative method, and its normalized values were equal to the relative widths of particle size distributions. The corresponding R_h_ values were calculated using the Stokes–Einstein equation.

### TEM observations

The TEM grid was pre-treated by glow discharge and then a drop of the sample (20 μL) was placed on the grid using a pipette. To fix the nanostructures in the studied mixtures, the sample-loaded TEM grid was freeze-dried using liquid nitrogen in a freeze-dryer. The sample was imaged using Cs-corrected (JEM–ARM200F, JEOL, USA) and high-resolution (JEM-2010, JEOL, USA) TEM instruments at the Jeonbuk National University (Republic of Korea).

### SAXS measurements

SAXS measurements were performed to obtain Fourier transforms of the hybrid nanostructures. To cover the Q-range (Q = (4π/λ) sin (θ/2) is the magnitude of the scattering vector and θ is the scattering angle) of 0.012–0.3 Å^−1^, the sample-to-detector distance was set to 2 m. The obtained data were normalized using the reduction software utilized at the 4C and 9A SAXS beamlines of the Pohang accelerator laboratory (POSTECH, Republic of Korea).

### SANS measurements

SANS measurements were carried out using the 40 m SANS instrument at HANARO (Korea Atomic Energy Research Institute (KAERI), republic of Korea). We used neutrons with a wavelength of λ = 7.49 Å. In order to cover the *q* range (where *q* = (4π/λ), sin(θ/2) is the magnitude of the scattering vector and θ is the scattering angle) from 0.0007 to 0.5 Å^−1^, three different sample to detector distances (SDDs), 1.16 m, 4.7 m, and 19.8 m (Lens) were used. The collected dataset was placed on an absolute scale using the direct beam flux method, and the calculations were performed using HANARO SANS data reduction software provided by KAERI. The SANS measurement was carried out in D_2_O to enhance the neutron scattering contrast.

## Supplementary Information

Supplementary Information.
