# Supplementary material for: Conformational control of two-dimensional gold nanoparticle arrays in a confined geometry within a vesicular wall
Source: Sci Rep. 2022 Mar 16;12:4548. doi: 10.1038/s41598-022-08607-0 (PMC8927576; doi:10.1038/s41598-022-08607-0)
Supplement: Supplementary file 1 — Supplementary Information. [file 41598_2022_8607_MOESM1_ESM.docx]

Supplementary Information

Conformational Control of Two-dimensional Gold Nanoparticle Arrays in a Confined Geometry within a Vesicular Wall

Jong Dae Jang, Hyuk-Jin Seo, Young-Jin Yoon, Soo-Hyung Choi, Young Soo Han, and Tae-Hwan Kim^*^


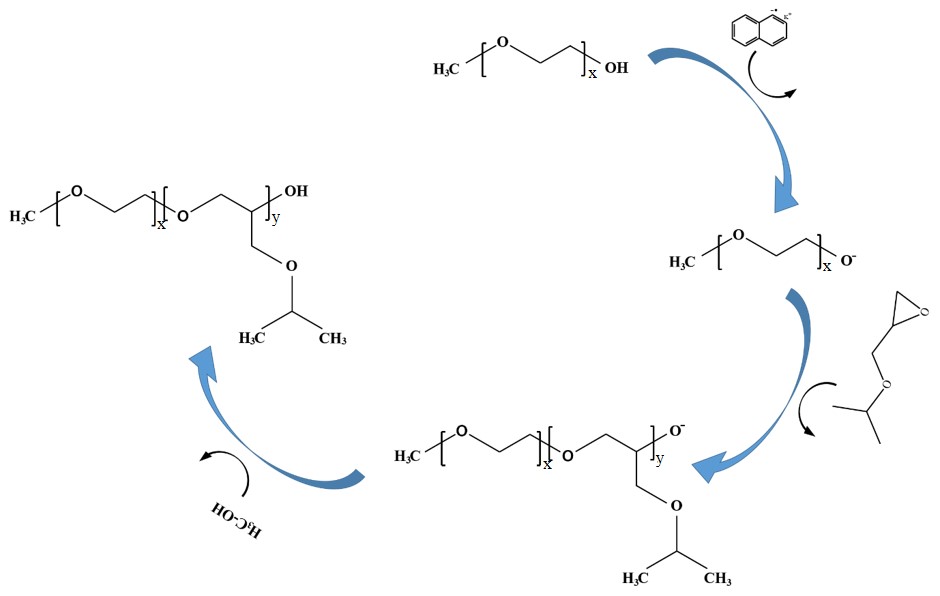
 **Fig. S1.** **Synthesis of the P(EG_x_-*b*-iPGE_y_) diblock copolymer.**


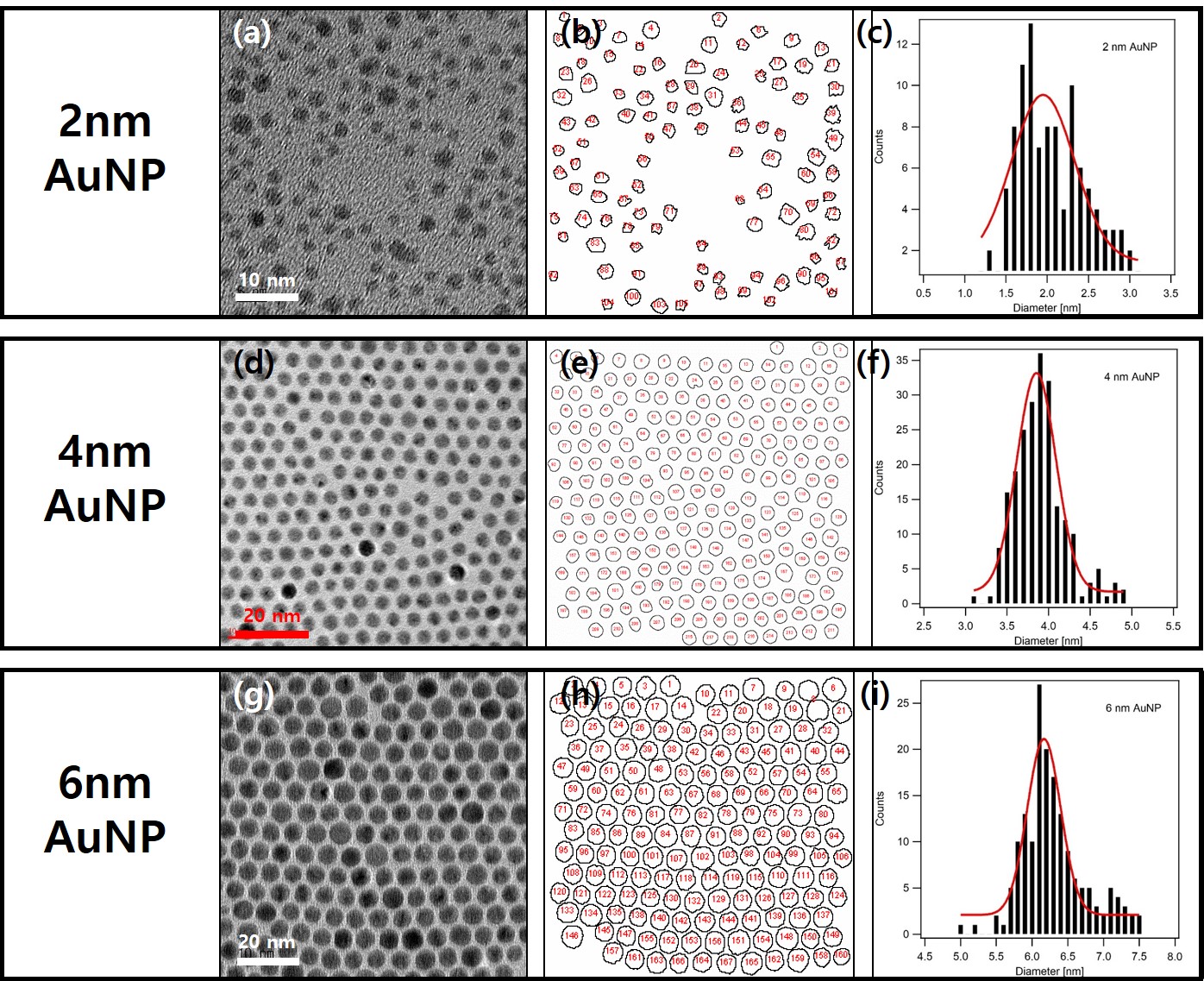
 **Fig. S2.** **Transmission electron microscopy** **(TEM) analysis of the synthesized AuNPs.** TEM images of (a) 2 nm, (d) 4 nm and (g) 6 nm calculate with a process of (b) 2 nm,(e) 4 nm and (h) 6 nm image counts to get the size distributions of (c) 2 nm,(f) 4 nm and (i) 6 nm, respectively.


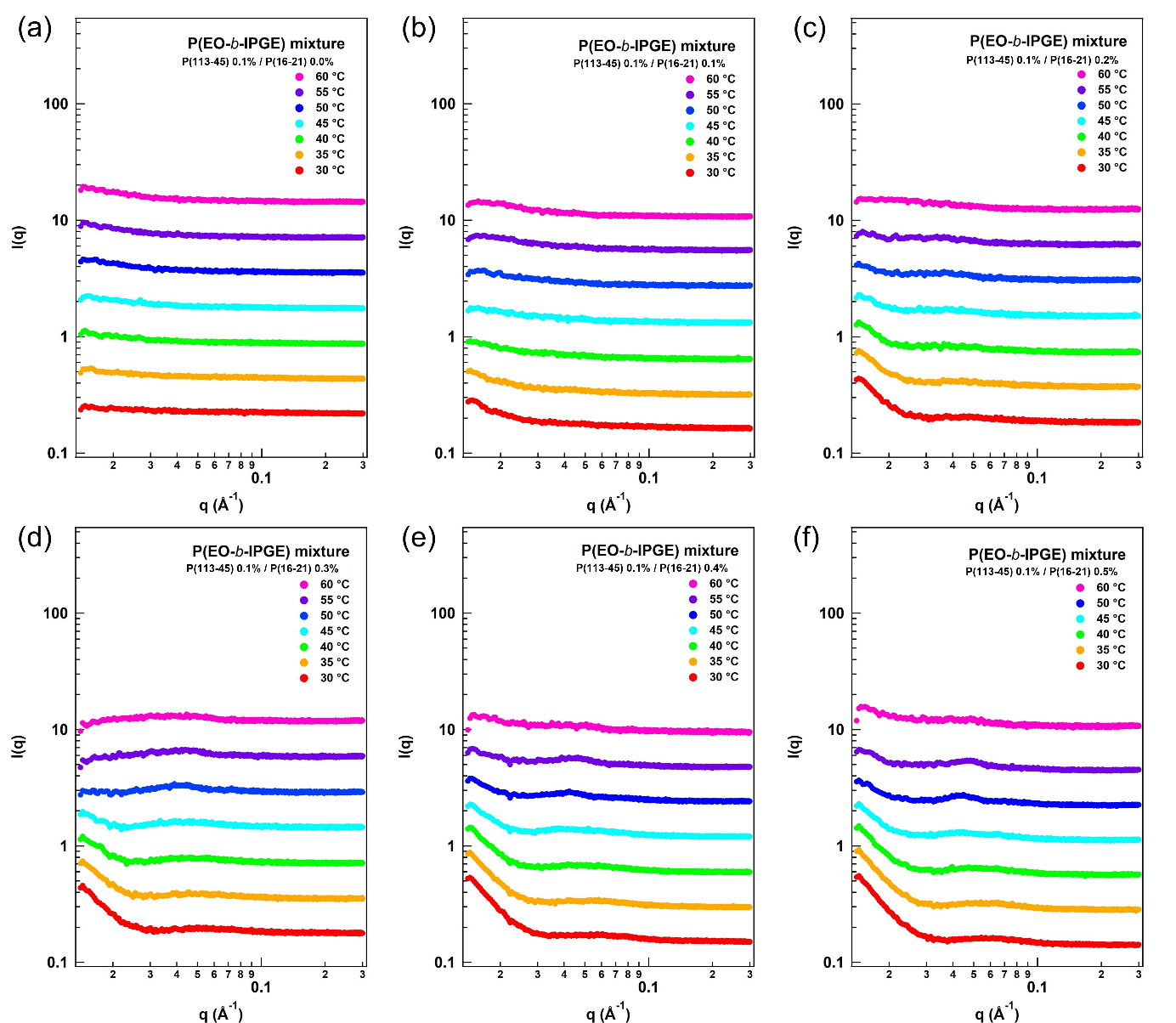


**Fig. S3. SAXS intensities of the P(113-45)/P(16-21) mixtures at varying the temperatures.** SAXS intensities of the P(113-45) 0.1 %/P(16-21) mixtures with the P(16-21) concentrations of (a) 0 %, (b) 0.1 %, (c) 0.2 %, (d) 0.3 %, (e) 0.4 %, and (f) 0.5 % in the water when the temperature increases from 30 ℃ to 60 ℃. SAXS intensities were vertically shifted for visual clarity.


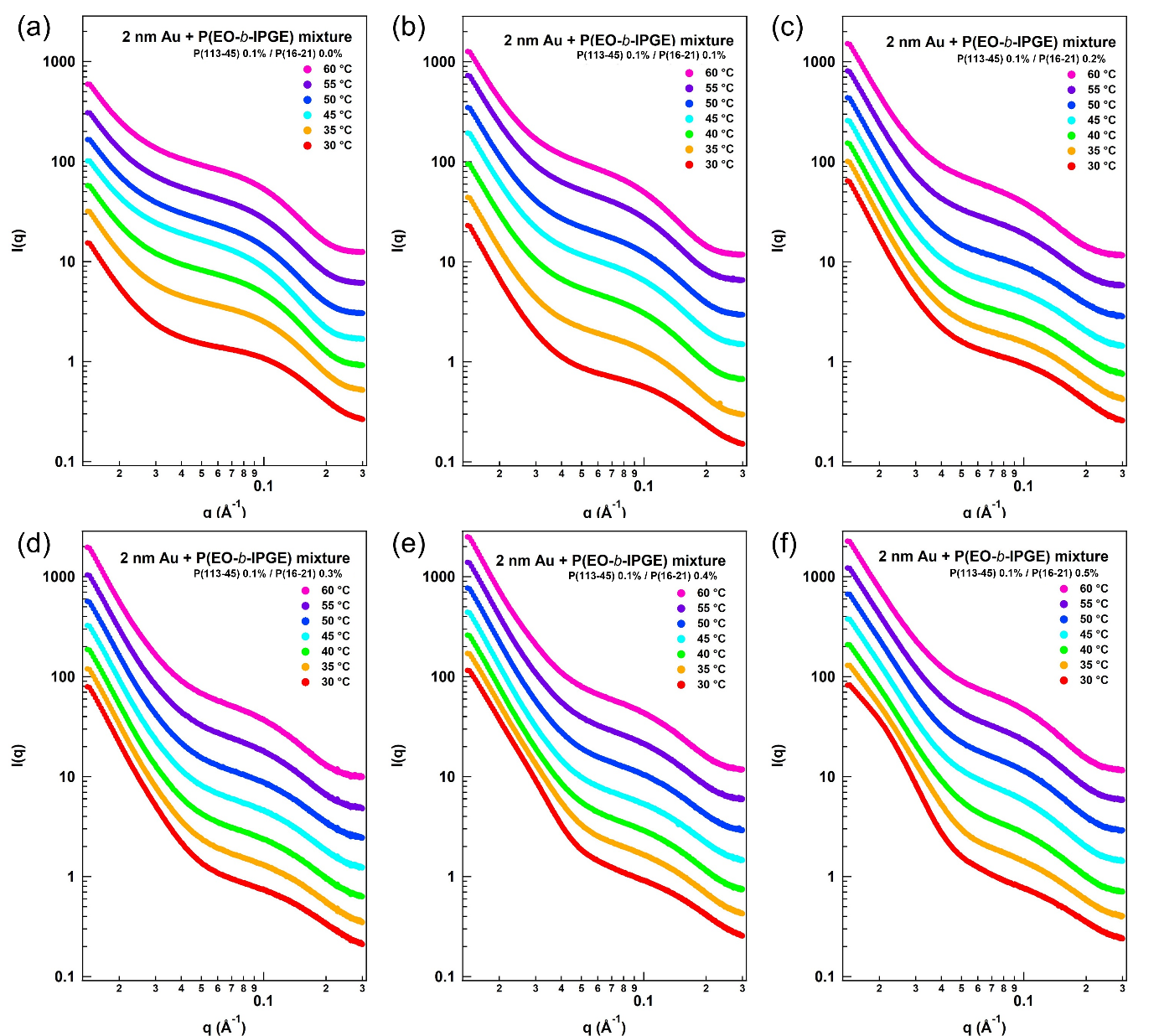


**Fig. S4. SAXS intensities of the 2 nm AuNPs-(113-45)/P(16-21) mixtures at varying the temperatures.** SAXS intensities of the P(113-45) 0.1 %/P(16-21) mixtures with the P(16-21) concentrations of (a) 0 %, (b) 0.1 %, (c) 0.2 %, (d) 0.3 %, (e) 0.4 %, and (f) 0.5 % in the water when the temperature increases from 30 ℃ to 60 ℃. SAXS intensities were vertically shifted for visual clarity.


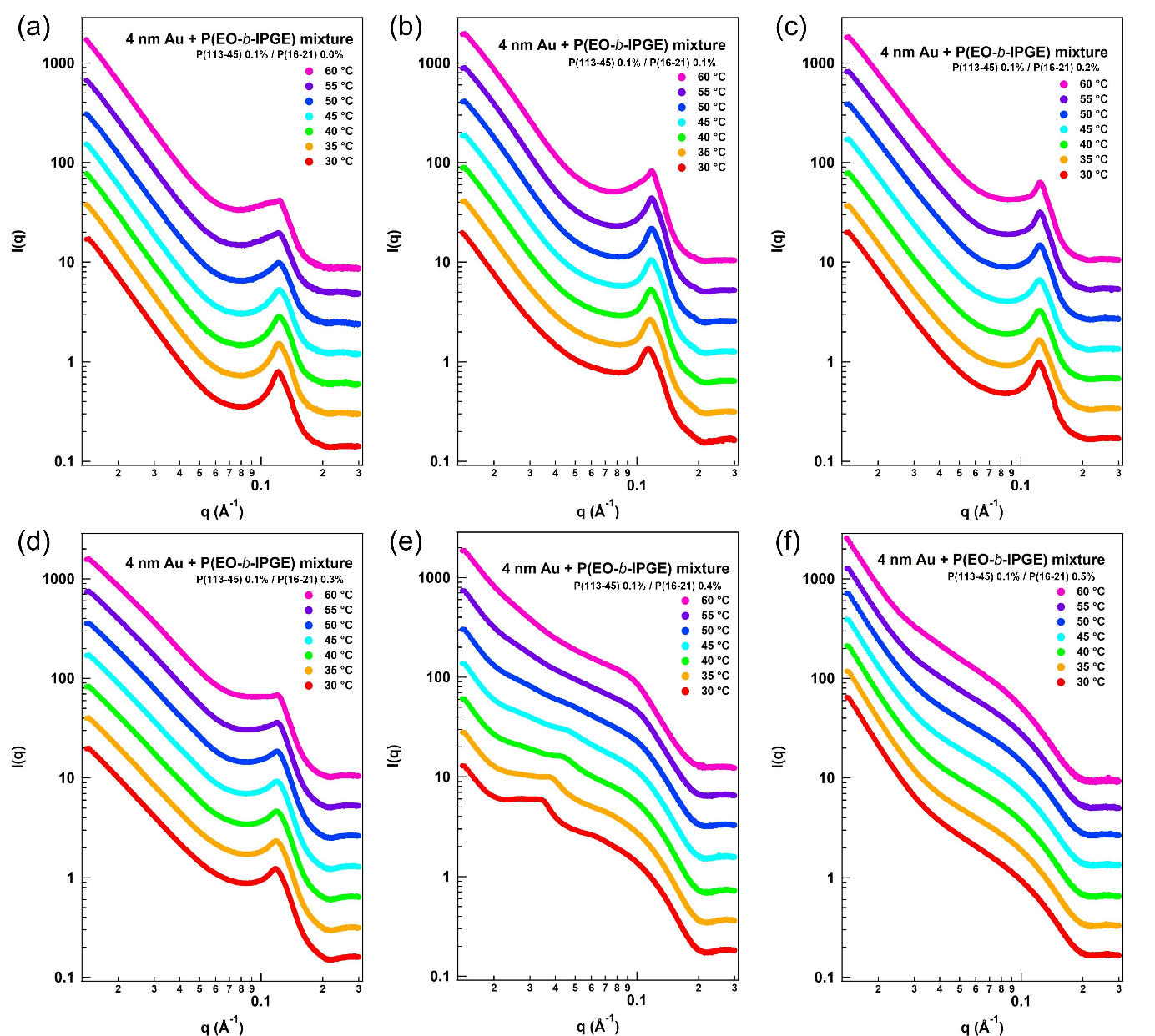


**Fig. S5. SAXS intensities of the 4 nm AuNPs-(113-45)/P(16-21) mixtures at varying the temperatures.** SAXS intensities of the P(113-45) 0.1 %/P(16-21) mixtures with the P(16-21) concentrations of (a) 0 %, (b) 0.1 %, (c) 0.2 %, (d) 0.3 %, (e) 0.4 %, and (f) 0.5 % in the water when the temperature increases from 30 ℃ to 60 ℃. SAXS intensities were vertically shifted for visual clarity.


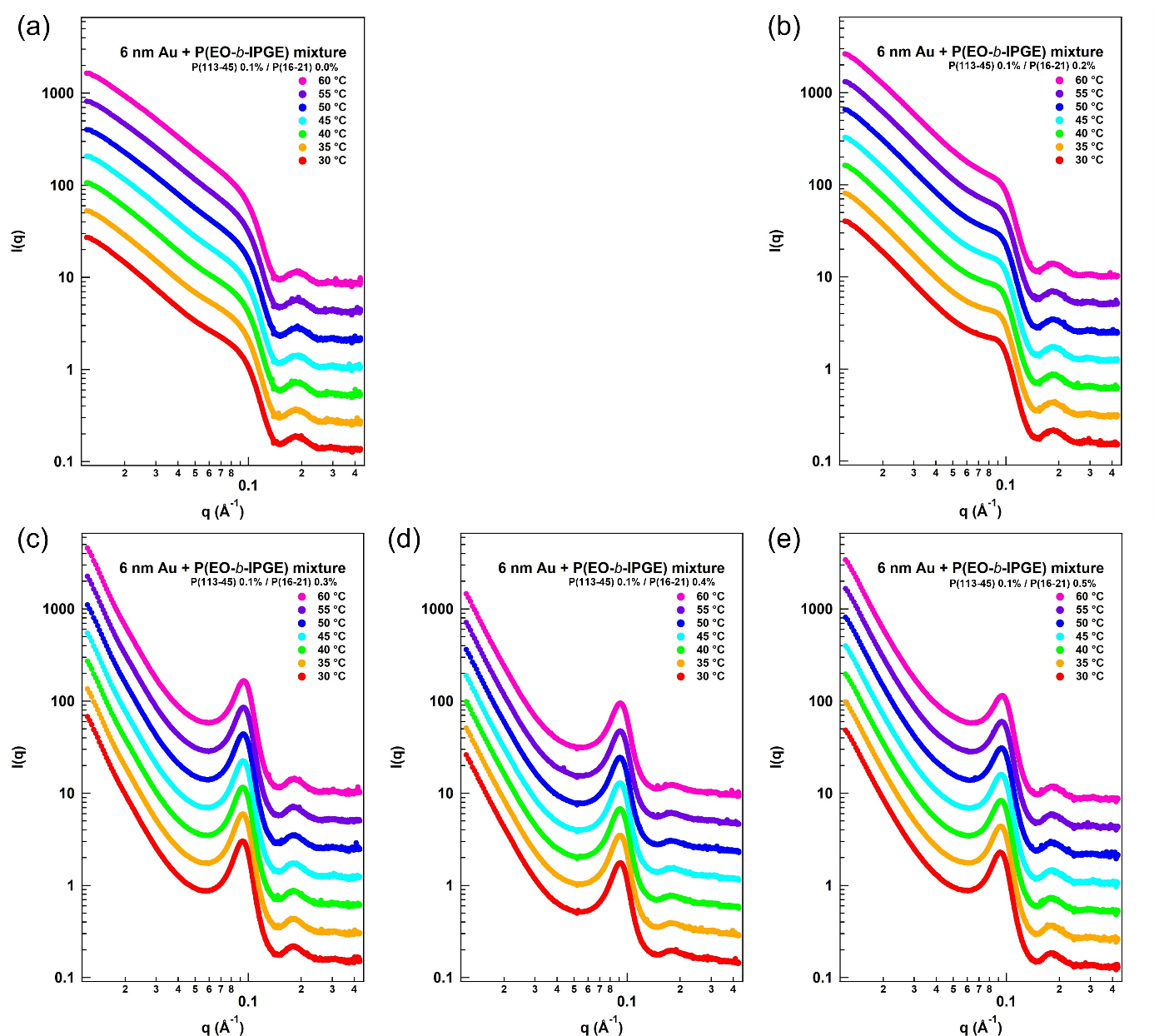


**Fig. S6. SAXS intensities of the 6 nm AuNPs-(113-45)/P(16-21) mixtures at varying the temperatures.** SAXS intensities of the P(113-45) 0.1 %/P(16-21) mixtures with the P(16-21) concentrations of (a) 0 %, (b) 0.2 %, (c) 0.3 %, (d) 0.4 %, and (e) 0.5 % in the water when the temperature increases from 30 ℃ to 60 ℃. SAXS intensities were vertically shifted for visual clarity.

**SANS measurement of AuNP/P(113-45)/P(16-21) mixture**

To support the nanostructure of AuNPs into the hydrophobic layer of polymer vesicle, the SANS measurement of AuNP/P(113-45)/P(16-21) mixture in D_2_O was performed (Fig. S7). The SANS intensity was analyzed by the form factor fitting method with a vesicle shape. The measured vesicular structure has a hydrophobic layer thickness of 3.5 nm, hydrophilic layer thickness 17.4 nm (2 layers), core diameter of 61.3 nm, and vesicle diameter of 103.1 nm, respectively.


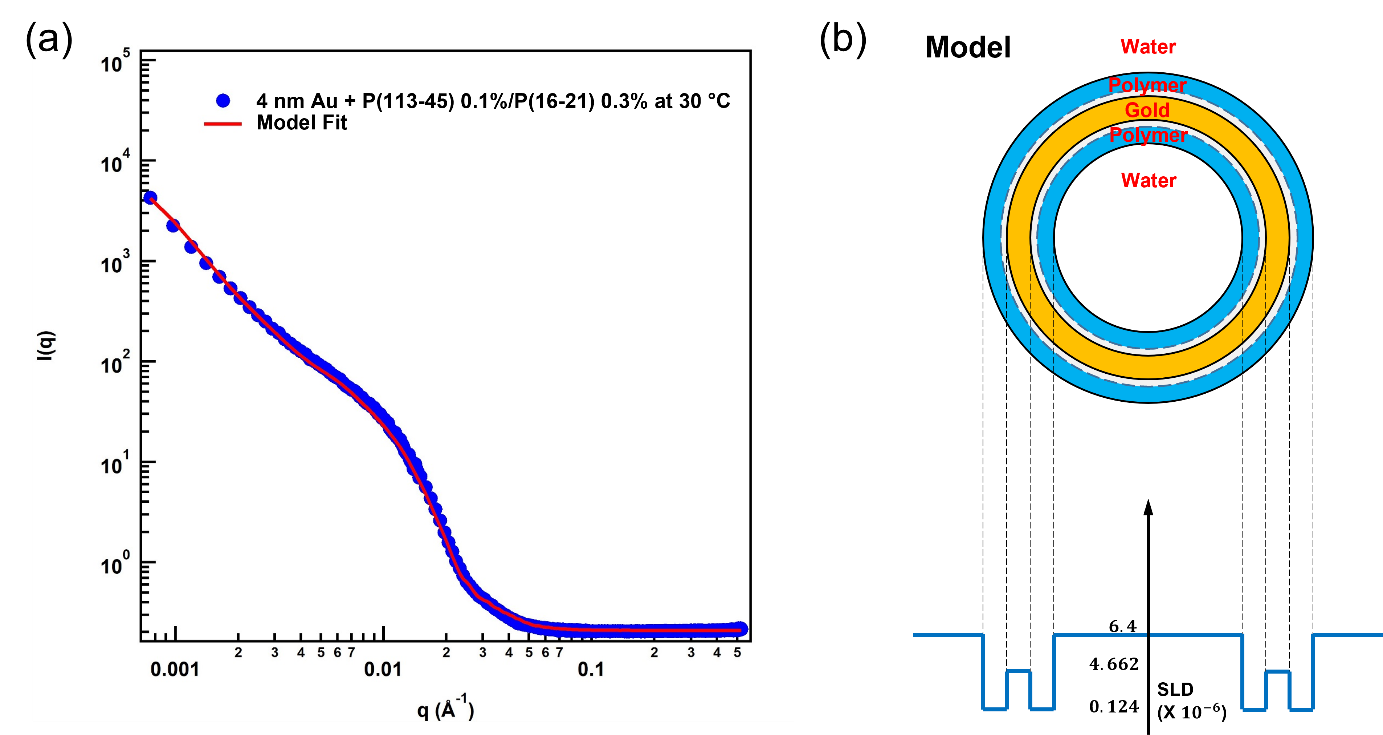


**Fig. S7. SANS intensity and model fit analysis of 4 nm AuNPs-(113-45) 0.1%/P(16-21) 0.3% mixture at 30 °C.** (a) SANS intensity and model fit of 4 nm AuNPs-(113-45) 0.1%/P(16-21) 0.3% mixtures at 30 °C. (b) Scattering length density (SLD) distribution model for the form factor analysis.
